# Report of Three Cases of Acute Cerebral Softening

**Published:** 1875-06-01

**Authors:** D. A. K. Steele

**Affiliations:** Late Resident Physician, Cook County Hospital


THE
### MEDICAL EXAMINER.
A
*Semi-Monthly Journal of Medical Sciences.*

EDITED BY N. S. DAVIS, M.D., AND F. H. DAVIS, M.D.

No. XI. CHICAGO, JUNE 1, 1875. VOL. XVI.

### Original Communications.

### REPORT OF THREE CASES OF ACUTE CEREBRAL SOFTENING.

BY D. A. K. STEELE, M.D., LATE RESIDENT PHYSICIAN, COOK COUNTY HOSPITAL.

THE following notes were taken while an *interne* in the County Hospital:

CASE I.—Michael M., age 41; cooper; native of Ireland; was admitted to the hospital May 19th, 1874. Stated that he was perfectly well until four weeks ago, when he noticed that his right hand and arm began to get weak. At the table, he could not hold his knife in the right hand. Sensation also became impaired. Four or five days ago, began to suffer with pains in the right chest. Symptoms have continued unchanged up to this date. States that for a number of years, he has been a moderate drinker, occasionally going on a spree, and then moderating down to his regular allowance. Will not admit that it has ever done him any harm.

*On Admission.*—Patient is a large, pretty well nourished man; tissues rather flabby; skin normal; tongue clean, but protruded to the right side slightly; appetite fair; bowels regular; urinates readily. Pulse 78; resp. 18. Complains of weakness in the right arm and hand, and pains in the right chest. Movements of arm apparently not much impaired, although there is a loss of muscular contractility. Was ordered tonics and a generous diet.

*May 21st.*—Had an apoplectic convulsion this morning, after which he lay in a semi-comatose condition until the 26th, when he died.

Post-mortem examination sixteen hours after death.

Incision from manubrium to umbilicus.

*Lungs.*—Healthy, with the exception of slight engorgement.

*Heart.*—Healthy.

*Liver.*—Normal in size, but upon the anterior surfce of the right lobe were found three small colloid cysts about the size of a filbert, also a small cretaceous mass about one-half inch in length, and one-fourth of an inch in diameter.

*Kidneys.*—Slightly congested; other abdominal organs normal.

On opening the cranium, found membranes healthy. On section of the cerebrum, found extensive softening of the whole central portion of the left hemisphere, the brain substance being reduced to a pulpiform mass; other portions of the brain apparently normal.

CASE II.—John N., aged 21; shoemaker; native of Denmark; admitted June 13th, 1874.

Patient states that he always enjoyed good health until four months ago, when he gradually began to have a feeling of coldness and numbness in the left arm and leg. Day by day became weak upon that side; lost his appetite; bowels continued pretty regular.

*On Admission.*—Find patient fairly nourished; no constitutional disturbance present. Find a partial loss of sensation, and of muscular power on the left side. Find a blowing murmur at the base of the heart, with the first sound. Was ordered potass. iodide grs. vij, and ol. morrhue ℥ ss. three times daily.

On examination of muscular power by the dynamometer, we find the index on left side 35°; on the right side 96°.

*July 11th.*—Left, 35°; right, 97°.

*July 14th.*—Left, 30°; right, 100°.

*July 17th.*—Left, 54°; right, 100°.

*July 13th.*—Patient complains of constant pain in the head, aggravated by any movement while lying down. For the past week, has experienced some difficulty in swallowing, fluids coming back through the nose. For the same length of time, has noticed that his articulation was becoming impaired. Could not speak certain words readily. Appetite very poor; bowels costive; tongue heavily coated with a whitish brown fur. Complains of dark spots floating before his eyes.

*July 14th.*—Tongue still furred. On protrusion, it deflects to the left.

*July 21st.*—Dynamometer on left side 31°; on right, 100°.

*July 27th.*—Left, 21°; right, 98°.

*July 28th.*—The test of electro-contractility and electro-sensibility, revealed diminished contractility of all the muscles of the upper extremities except the pectoral muscles. Electro-contractility diminished in the legs, but sensibility good.

*August 7th.*—Paralysis is becoming more marked. Patient sweats profusely. Bowels obstinately constipated. Pulse 88; temp. 98; resp. 28. Pupils widely dilated. Passes urine and fæces involuntarily. Is wildly delirious, talking in an incoherent semi-idiotic manner; rapidly growing weaker; unable to leave the bed; face assuming a dull leaden expression, constantly covered with a cold perspiration. Paralysis of the pharyngeal muscles prevents intelligent articulation.

*August 9th.*—Died apparently from a gradual paralysis of the vital organs. Had no convulsions preceding death —passed away quietly.

*Sectio - cadaveris.* — Twenty - four hours after death, body presents an exsanguinated appearance. Lungs, healthy. Heart, small. Valves, normal; right side distended with venous blood. Liver and spleen, small and congested. Kidneys, normal in size, but intensely congested. In fact, all the viscera presented a hyperæmic appearance.

On removing the calvarium, the dura mater seemed slightly thickened. A small amount of serum at base of brain; superficial vessels of brain distended with blood. On making section of the brain, found in left hemisphere all of the anterior and superior portion of the gray matter healthy. Lower portion of gray matter soft and diffluent. In the anterior lobe of left hemisphere, the white matter is healthy, but the posterior and inferior portions are soft and diffluent. In the right hemisphere, gray matter healthy, superiorly. Inferior portion of gray matter and all the white matter, soft and disorganized. Cerebellum softened, especially on the left side; pons varolii in the same condition. Spinal cord, healthy.

CASE III.—N——, aged 28; laborer; Poland; admitted August 24th, 1874.

Having no interpreter, and the patient being unable to speak much English, it is impossible to get an accurate history of his ailment.

It seems he has been sick about six weeks; was working in a lumber yard previous to this time, when he caught cold; has had several chills; thought he was sun-struck. Appetite has been poor; bowels costive; and he has complained of pains in the lower extremities ever since.

*August 25th.* — Has retention of urine, requiring the daily use of catheter. Urine highly ammoniacal. Bowels constipated. Was ordered an enema.

*August 26th.*—Again catheterized; slept some last night. On the chest and abdomen, find a sudaminous eruption; cuticle in places desquamating. Circulation exceedingly sluggish. Patient has a dusky hue. Pulse 132; temp. 97; resp. 18. Bronchial breathing in the orifices of both lungs; most marked in the left.

P.M.—Bowels again moved by an enema; discharge very offensive.

*August 27th.*—For the past week, patient has had paralysis of the inferior extremities. Has been unable to walk. Commenced with a feeling of stiffness and pains in the legs, and gradual loss of power. Has slight difficulty in swallowing. This morning, find face flushed; tongue slightly furred; pulse 120; temp. 98½; resp. 16. Legs are almost completely paralyzed; sensation somewhat impaired. The muscles are tender, on pressure, particularly in the legs. Ordered pot. iodide, gr. vi, tr. digitalis, m xx, ter die; also, to have catheter used every six hours, and bowels to be kept free by enema.

*August 28th.* — Tongue not deflected, but tremulous. Complains of more pain in the legs. There is entire loss of muscular power in the arms. Pulse 120; temp. 99; resp. 20. Left side of face seems drawn; is unable to close the left eye. In winking, can scarcely move the left eyelid at all. Movements of the eyes are symmetrical.

*August 29th.*—Pharyngeal muscles becoming more impaired; slept poorly last night; no improvement in any of the symptoms.

*September 1st.* — Patient's friends took him home, as dissolution was rapidly approaching.

*September 2d.* — Learned from friends that patient died to-day, but was unable to obtain a post-mortem examination.

